# Radiosynthesis and Biological Investigation of a Novel Fluorine-18 Labeled Benzoimidazotriazine-Based Radioligand for the Imaging of Phosphodiesterase 2A with Positron Emission Tomography

**DOI:** 10.3390/molecules24224149

**Published:** 2019-11-15

**Authors:** Rien Ritawidya, Barbara Wenzel, Rodrigo Teodoro, Magali Toussaint, Mathias Kranz, Winnie Deuther-Conrad, Sladjana Dukic-Stefanovic, Friedrich-Alexander Ludwig, Matthias Scheunemann, Peter Brust

**Affiliations:** 1 Helmholtz-Zentrum Dresden-Rossendorf, Institute of Radiopharmaceutical Cancer Research, Research Site Leipzig, Department of Neuroradiopharmaceuticals, 04318 Leipzig, Germany; b.wenzel@hzdr.de (B.W.); r.teodoro@hzdr.de (R.T.); m.toussaint@hzdr.de (M.T.); w.deuther-conrad@hzdr.de (W.D.-C.); s.dukic-stefanovic@hzdr.de (S.D.-S.); f.ludwig@hzdr.de (F.-A.L.); m.scheunemann@hzdr.de (M.S.); p.brust@hzdr.de (P.B.); 2 Center for Radioisotope and Radiopharmaceutical Technology, National Nuclear Energy Agency (BATAN), Puspiptek Area, Serpong, South Tangerang 15314, Indonesia; 3 Tromsø PET Center, University Hospital of North Norway, 9009 Tromsø, Norway; Mathias.Kranz@unn.no; 4 Nuclear Medicine and Radiation Biology Research Group, The Arctic University of Norway, 9009 Tromsø, Norway

**Keywords:** cyclic nucleotide phosphodiesterase, PDE2A radioligand, nitro-precursor, fluorine-18, in vitro autoradiography, PET imaging

## Abstract

A specific radioligand for the imaging of cyclic nucleotide phosphodiesterase 2A (PDE2A) via positron emission tomography (PET) would be helpful for research on the physiology and disease-related changes in the expression of this enzyme in the brain. In this report, the radiosynthesis of a novel PDE2A radioligand and the subsequent biological evaluation were described. Our prospective compound 1-(2-chloro-5-methoxy phenyl)-8-(2-fluoropyridin-4-yl)-3- methylbenzo[e]imidazo[5,1-c][1,2,4]triazine, benzoimidazotriazine (**BIT1**) (IC_50_ PDE2A = 3.33 nM; 16-fold selectivity over PDE10A) was fluorine-18 labeled via aromatic nucleophilic substitution of the corresponding nitro precursor using the K[^18^F]F-K_2.2.2_-carbonate complex system. The new radioligand [^18^F]**BIT1** was obtained with a high radiochemical yield (54 ± 2%, n = 3), a high radiochemical purity (≥99%), and high molar activities (155–175 GBq/μmol, n = 3). In vitro autoradiography on pig brain cryosections exhibited a heterogeneous spatial distribution of [^18^F]**BIT1** corresponding to the known pattern of expression of PDE2A. The investigation of in vivo metabolism of [^18^F]**BIT1** in a mouse revealed sufficient metabolic stability. PET studies in mouse exhibited a moderate brain uptake of [^18^F]**BIT1** with a maximum standardized uptake value of ~0.7 at 5 min p.i. However, in vivo blocking studies revealed a non-target specific binding of [^18^F]**BIT1**. Therefore, further structural modifications are needed to improve target selectivity.

## 1. Introduction

The cyclic nucleotide phosphodiesterases (PDEs) are a superfamily of enzymes catalyzing the hydrolysis of the intracellular secondary messengers, cyclic adenosine monophosphate (cAMP) and cyclic guanosine monophosphate (cGMP) [1,2,3]. These secondary messengers are involved in a great variety of cellular functions associated with normal and pathophysiological processes in the brain and periphery [3,4,5,6].

The 11 family members of PDEs are encoded by 21 genes and classified by their substrate specificity [3,7]. PDE 4, 7, and 8 are only cAMP-specific, PDE 5, 6, and 9 are cGMP-specific, and the remaining PDE 1, 2, 3, 10, and 11 hydrolyze both substrates [3,6,7].

The dual-substrate enzyme PDE2A is abundantly expressed in brain, in particular, in caudate, nucleus accumbens, cortex, hippocampus [3,8,9], amygdala [10,11], substantia nigra, as well as olfactory tubercle [11,12], while the expression in the midbrain, hindbrain, and cerebellum is comparatively low [8,11,12]. This specific distribution indicates a role of PDE2A in the modulation of complex neuronal processes, such as learning, concentration, memory, emotion, depression, anxiety, and CNS related disorder [10,13,14]. The pharmacological inhibition of PDE2A has been evaluated in preclinical studies, thus suggesting PDE2A inhibitors as a potential treatment for neurodegenerative diseases, such as Alzheimer’s disease, schizophrenia, and dementia [15,16]. PDE2A inhibition has the potential to prolong the duration of cAMP- and cGMP-dependent signaling pathways, eventually improving neural plasticity and memory function [3,13,17].

Positron emission tomography (PET) is a molecular imaging modality that enables the visualization, characterization, and measurement of molecular targets and biochemical processes in living systems [18]. Accordingly, a PDE2A specific radiotracer would allow quantification of PDE2A expression, as well as disease-related changes thereof.

**Figure 1:**
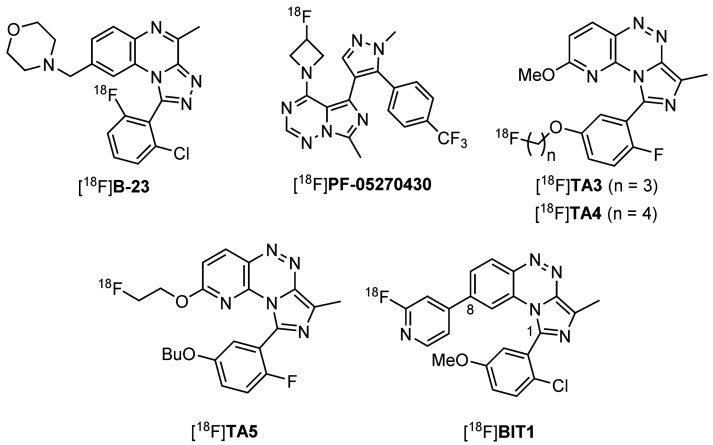
Selected phosphodiesterase 2A (PDE2A) PET radioligands [10,19,20,23,24,25].

The so far most developed PDE2A radioligands are shown in Figure 1. The first highly potent PDE2A compound [^18^F]**B-23** (IC_50_ hPDE2A = 1 nM; IC_50_ rPDE10A = 11 nM) was developed by Janssen Pharmaceutica NV (Beerse, Belgium) [10,19]. Biodistribution and microPET imaging studies in rats demonstrated high uptake of activity in the striatum; however, brain-penetrating radio-metabolites limited further evaluation. Pfizer Inc. (New York, NY, USA) also reported on a highly affine PDE2A radioligand, [^18^F]**PF-05270430** (IC_50_ hPDE2A = 0.5 nM; IC_50_ hPDE10A >3000 nM) [10,20], which has been evaluated in monkeys [20] and translated to clinical trials [21,22]. [^18^F]**PF-05270430** showed high target-specific accumulation in the putamen, caudate, and nucleus-accumbens, as well as good metabolic stability and a favorable kinetic profile, pointing out [^18^F]**PF-05270430** as a promising PDE2A PET ligand [21]. However, using the cerebellum as a reference region, the estimated binding potential of [^18^F]**PF-05270430** was low, and the authors concluded that further studies are required to validate the suitability of the cerebellum as a reference region [21].

In parallel to Pfizer, our group developed further PDE2A radioligands on the basis of pyridoimidazotriazine. The recently published compounds [^18^F]**TA3** (IC_50_ PDE2A = 11.4 nM; IC_50_ PDE10A = 318 nM) and [^18^F]**TA4** (IC_50_ PDE2A = 7.3 nM; IC_50_ PDE10A = 913 nM) (Figure 1) were characterized by high potency and selectivity towards PDE2A [24]. Notably, both radiotracers were found to be suitable radioligands for in vitro imaging of PDE2A; however, in vivo metabolism studies revealed a high fraction of polar radio-metabolites in the brain limiting their use for in vivo PDE2A imaging. The latest radioligand out of this series, [^18^F]**TA5** ((IC_50_ PDE2A = 3.0 nM; IC_50_ PDE10A >1000 nM, Figure 1), exhibited the highest potency towards PDE2A and selectivity over PDE10A. However, autoradiographic studies of [^18^F]**TA5** showed a homogenous and non-displaceable binding in rat and pig brain cryosections, indicating an insufficient specificity of this radioligand [25]. In addition, [^18^F]**TA5** is degraded extremely fast in the mouse. Therefore, we decided to perform further structural modifications to develop fluorine-18 labeled PET tracers with improved metabolic stability for the molecular imaging of PDE2A.

In our continuous effort to develop fluorine-18 labeled PET tracers dedicated to molecular imaging of PDE2A, we selected the benzoimidazotriazine (BIT) scaffold (Figure 1) as lead structure by replacing the pyrido ring of the **TA** compounds with a benzo ring [26,27]. As a first result, we very recently reported on the synthesis and inhibitory potency of nine new fluorinated derivatives based on this BIT scaffold [28]. In order to increase the metabolic stability of the corresponding PDE2A radioligands, the fluorine was introduced by substituting a fluoropyridine ring at the benzene moiety. Out of this series, derivative **BIT1** (Figure 1) was selected as the most suitable candidate for ^18^F-labeling and biological investigation (IC_50_ PDE2A = 3.33 nM; 16-fold selectivity over PDE10A) [28].

Herein, we reported on the development and the evaluation of [^18^F]**BIT1**, including the synthesis of the corresponding nitro precursor, the manual radiosynthesis, and the transfer to an automated synthesis module, and the subsequent in vitro and in vivo investigations.

## 2. Results and Discussion

### 2.1. Precursor Synthesis and Radiochemistry

#### 2.1.1. Synthesis of the Labeling Precursor **5**

**Scheme 1:**
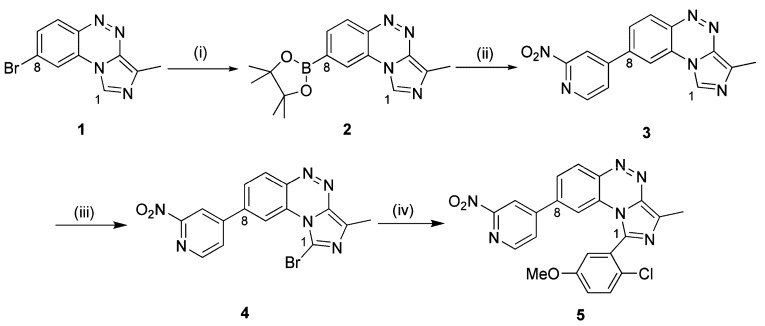
Synthesis of precursor **5**. Reagents and conditions: (i) Bis(pinacolato)diboron [Pd(dppf)Cl_2_], KOAc, 2-methyltetrahydrofuran (2-MeTHF), 100 °C (6 h), 85 °C (12 h), 85%; (ii) 4-bromo-2-nitropyridine, K_2_CO_3_, Pd(PPh_3_)_4_, 1,2-dimethoxyethane, 63%; (iii) N-bromo-succinimide (NBS), acetonitrile (MeCN), room temperature (RT), 4–6 h, 97%; (iv) 2-chloro-5-methoxy-phenyl-1-yl-boronic acid, K_2_CO_3_, [Pd(PPh_3_)_4_], 1,4-dioxane/H_2_O (4:1), reflux, 1–2 d, 68%.

The synthesis of the reference compound **BIT1** has been reported previously [28]. For an efficient radiosynthesis of [^18^F]**BIT1**, we selected the nitro precursor **5** because of its higher reactivity to nucleophilic aromatic substitutions in comparison to a bromo-substituted precursor [29,30]. As depicted in Scheme 1, **5** was prepared in four steps starting from the BIT key intermediate **1 [28]**. Firstly, Miyaura borylation of **1** with bis(pinacolato)diboron, using potassium acetate and [Pd(dppf)Cl_2_] as base and catalyst, afforded the pinacol boronic ester **2** in a satisfactory yield of 85% [31]. By palladium-catalyzed Suzuki coupling with 4-bromo-2-nitropyridine, compound **3** was obtained in 63% yield. This 4-bromo-2-nitropyridine was synthesized according to a slightly modified procedure, already described in the literature [32]. Thereafter, the bromination at 1-position of the imidazole ring using *N*-bromo-succinimide (NBS) afforded compound **4** in an excellent yield of 97%. Finally, the subsequent Suzuki coupling with 2-chloro-5-methoxy-phenyl boronic acid gave the nitro precursor **5** in 68% yield (NMR spectrums of precursor **5** see in the Supplementary Materials).

#### 2.1.2. Radiosynthesis and Characterization of [^18^F]**BIT1**

##### Manual Radiosynthesis

The new PDE2A radioligand [^18^F]**BIT1** was prepared by heteroaromatic nucleophilic substitution of **5** using a classical anhydrous K[^18^F]F-K_2.2.2_ carbonate complex. The radiolabeling process of [^18^F]**BIT1** was carried out under thermal heating applying a constant amount of 1.0 mg of precursor **5** by using different (i) polar aprotic solvents dimethylsulfoxide (DMSO), *N,N*-dimethylformamide (DMF), and acetonitrile (MeCN), (ii) temperatures, and (iii) reaction times.

**Figure 2:**
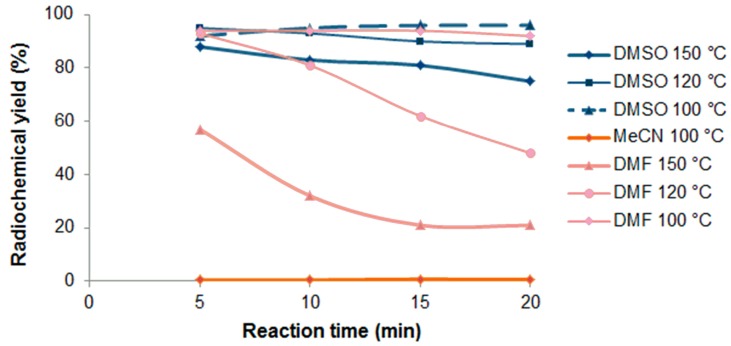
The radiochemical yield of [^18^F]**BIT1** depending on reaction time, temperature, and solvent. (Conditions: K[^18^F]F-K_2.2.2_, thermal heating, 1.0 mg of **5**). BIT, benzoimidazotriazine.

As shown in Figure 2, after 5 min (min) reaction time, a radiochemical yield (RCY) of ~57% was obtained when DMF was used at 150 °C. According to radio-TLC, besides [^18^F]fluoride, three radioactive by-products were observed, which accounted for 10% of total radioactivity. When the temperature was reduced to 120 °C, an increase of the RCY up to 93% was observed at this early time point. However, the RCY decreased with increasing the reaction time, indicating a decomposition of [^18^F]**BIT1** under these conditions. The highest RCY in DMF (~94%) was achieved when the reaction temperature was further reduced to 100 °C. At this temperature, the product remained stable over 20 min reaction time. By contrast, almost no radiofluorination could be observed when MeCN was used (RCY ≤1%). With DMSO as a solvent, high RCYs (>85%) were obtained at 150 °C and 120 °C, which remained constant up to 20 min reaction time in contrast to the findings with DMF. A further decrease to 100 °C resulted in an increase of the RCY (>95%) after 10 min of reaction time. The precursor **5** was stable throughout the time of analysis, as proven by HPLC (data not shown). Based on these results, DMSO was selected for the production of [^18^F]**BIT1**.

Due to the similarity of the chromatographic behavior of the nitro precursor and the corresponding ^18^F-radiotracer, the use of a low amount of precursor is beneficial for subsequent isolation of the radiotracer via semi-preparative HPLC [33,34]. Accordingly, the further reduction of the amount of the nitro precursor **5** up to 0.5 mg was investigated, achieving an excellent RCY of ≥95% (DMSO, 100 °C, 5 min reaction time). The selection of a suitable column for semi-preparative HPLC was also somewhat crucial in order to have a separation of the radiotracer and its nitro precursor in a reasonable time [33]. According to our previous experiences [33], a slightly polar C18 phase turned out to be most appropriate for the isolation of [^18^F]**BIT1** using a mixture of MeCN/water and ammonium acetate as a buffer.

[^18^F]**BIT1** was successfully isolated under the aforementioned conditions and further purified by solid-phase extraction (SPE). After formulation in 0.9% saline containing 10% ethanol, [^18^F]**BIT1** was obtained with an RCY of 38% (decay corrected to the end of the bombardment, EOB), and molar activities in the range of 38 GBq/μmol (at the end of synthesis, EOS).

##### Automated Radiosynthesis of [^18^F]**BIT1**

**Scheme 2:**
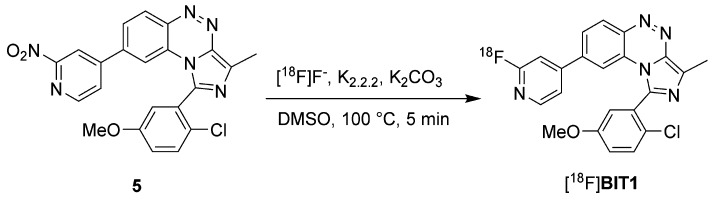
^18^F-labeling of [^18^F]**BIT1.**

**Figure 3:**
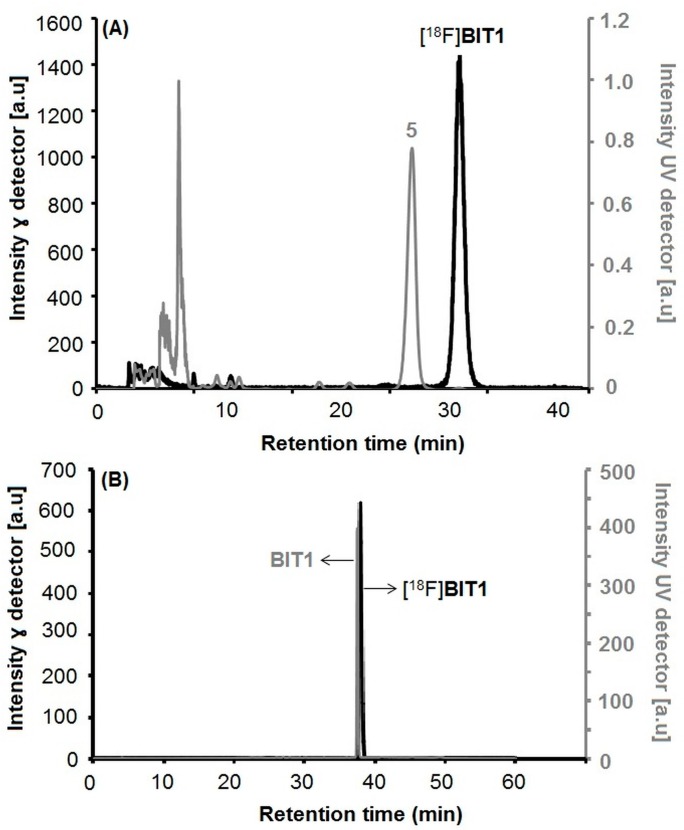
(**A**) Semi-preparative radio- and UV-HPLC chromatograms of [^18^F]**BIT1** (conditions: Reprosil-Pur C18-AQ, 250 × 10 mm, 46% MeCN/20 mM NH_4_OAc_aq_., 5.5 mL/min); (**B**) Analytical radio- and UV-HPLC chromatograms of the final product of [^18^F]**BIT1** spiked with the non-radioactive reference **BIT1** (conditions: Reprosil-Pur C18-AQ, 250 × 4.6 mm, gradient system with eluent mixture of MeCN/20 mM NH_4_OAc_aq_., 1.0 mL/min).

By using the most appropriate conditions of the manual procedure, the automated radiosynthesis of [^18^F]**BIT1** (Scheme 2) was established using a TRACERlab FX2 N synthesis module (GE Healthcare, Waukesha, WI, USA). The detailed configuration is shown in theexperimental part. Briefly, the [^18^F]F^-^ was firstly trapped on an anion exchange cartridge and then eluted into the reactor using an aqueous potassium carbonate solution. The reaction took place with the [^18^F]F^-^/K_2.2.2._/K_2_CO_3_ system and the nitro precursor **5** (0.5 mg) in DMSO at 100 °C for 5 min. After the isolation of [^18^F]**BIT1** via semi-preparative RP-HPLC (Figure 3A), the product was purified via SPE on an RP cartridge and formulated in sterile isotonic saline containing 10% of EtOH. The total synthesis time of [^18^F]**BIT1** was approximately 75 min. Analytical radio- and UV-HPLC of the final product, spiked with the non-labeled reference compound **BIT1**, confirmed the identity of [^18^F]**BIT1** (Figure 3B). Finally, the radiotracer was obtained with a radiochemical purity of ≥99%, an RCY of 54 ± 2% (EOB, n = 3), and molar activities in the range of 155–175 GBq/μmol (EOS, n = 3).

##### In vitro Stability and Lipophilicity of [^18^F]**BIT1**

The radioligand [^18^F]**BIT1** was stable (radiochemical purity ≥99%) in phosphate-buffered saline (PBS), pig plasma, *n*-octanol, as well as in the saline formulation containing 10% ethanol at 40 °C for up to 1 h. The lipophilicity was determined by the shake-flask method in the *n*-octanol/PBS system. With a logD_7.4_ value of 1.81 ± 0.05, [^18^F]**BIT1** falls within the range of radiotracers with optimal brain passive diffusion [35,36,37]. However, the calculated coefficient distribution value (ACD/Labs, Version 12.0, Advanced Chemistry Development, Inc.) displayed value of 4.03. The significant deviations between the calculation and experimental methods were observed in particular when the pattern of connectivity and non-bonded intramolecular interactions are not included in the applied database [38,39,40,41]. Moreover, the big discrepancy between the experimental and the calculated logD_7.4_ value was already observed for our PDE2 tracer [^18^F]**TA5** and has been previously discussed [25]. It is assumed that the experimentally determined higher hydrophilicity of [^18^F]**TA5** might be due to the solvation effect related to hydrogen bonding and ionization of the radioligand in the aqueous buffered system [25,35]. Whereas with the software-based determination, this effect may be underestimated [25]. Therefore, the calculated logD value is often higher than the experimentally determined value [25,35]. Since **TA5** and **BIT1** are rather structurally similar, we assume the apparent strong discrepancy of logD for [^18^F]**BIT1** might also be caused by the same reasons as those for [^18^F]**TA5**.

### 2.2. In Vitro and In Vivo Characterization of [^18^F]**BIT1**

#### 2.2.1. In Vitro Autoradiography of [^18^F]**BIT1**

**Figure 4:**
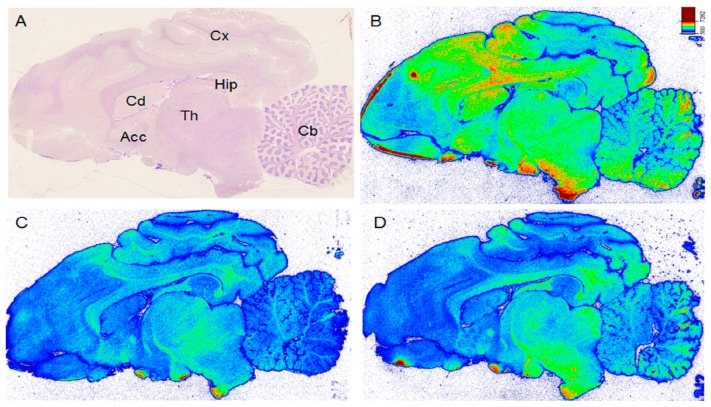
In vitro autoradiography of [^18^F]**BIT1** in pig brain slices, (**A**) Nissl staining, (**B**) Total binding of 1.72 nM of [^18^F]**BIT1**, (**C**) Blocking studies (10 µM of **TA1**), (**D**) Self-blocking (10 µM of **BIT1**). Abbreviations: (Acb: nucleus accumbens; Cb: cerebellum; Cd: nucleus caudatus; Cx: cortex; Hip: hippocampus; Th: thalamus).

To investigate the distribution of binding sites of [^18^F]**BIT1** in the brain, in vitro autoradiographic studies using cryosections of pig brain were performed. As depicted in Figure 4B, the distribution pattern of [^18^F]**BIT1** corresponds to the known spatial distribution of PDE2A with a high density of binding sites in the caudate nucleus (Cd), nucleus accumbens (Acb), cortex (Cx), and hippocampus (Hip) (Nissl staining of the corresponding slice is shown in Figure 4A). However, [^18^F]**BIT1** also binds to the non-PDE2A specific regions cerebellum (Cb) and thalamus (Th), indicating binding of [^18^F]**BIT1** to other targets.

To verify these findings, blocking studies with 10 µM of **TA1** (a potent PDE2 ligand) [42] (Figure 4C) and **BIT1** (Figure 4D) were performed. The decrease of [^18^F]**BIT1** binding of ~50% and ~30% in PDE2A specific regions Cd and Acb, respectively, observed for both **TA1** and **BIT1** indicated in vitro specificity of the radiotracer. However, the simultaneous decrease of [^18^F]**BIT1** binding in the range of 20–30% in Cb by **TA1**, as well as **BIT1**, suggested further non-specific binding of the radiotracer. We hypothesized the high non-specific binding of [^18^F]**BIT1** could be related to the moderate selectivity of **BIT1** over PDE10A. Accordingly, these results limited the suitability of [^18^F]**BIT1** for in vitro molecular imaging of PDE2A.

#### 2.2.2. In Vivo Metabolism of [^18^F]**BIT1**

**Figure 5:**
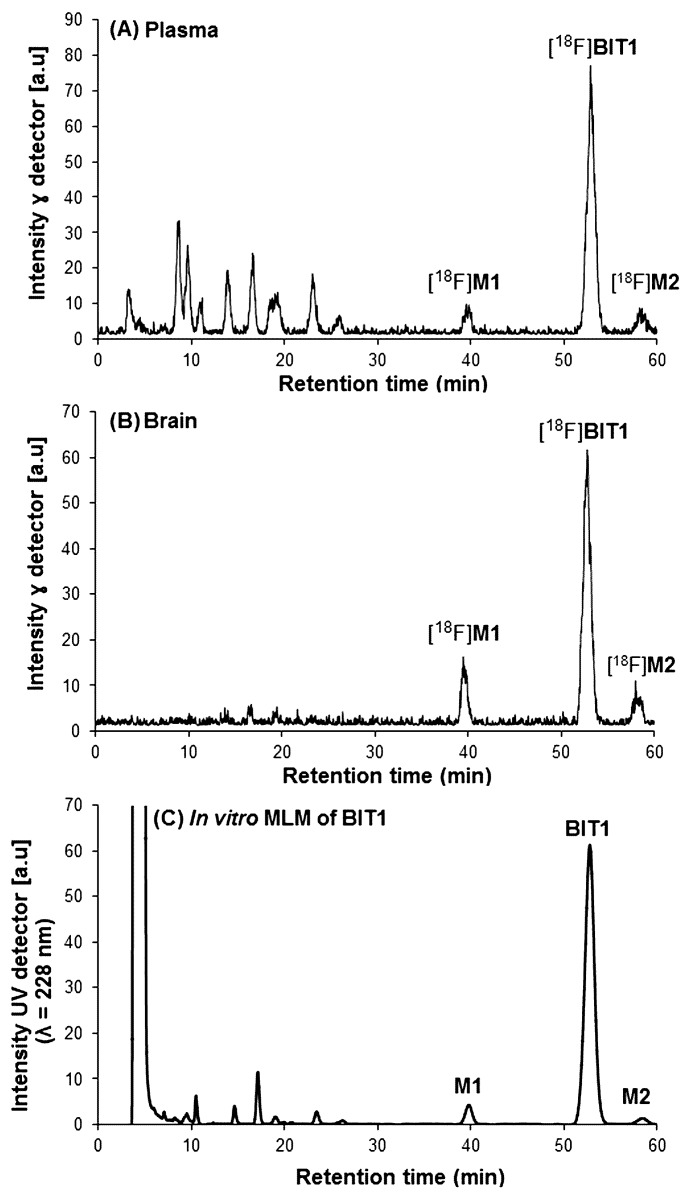
Representative analytical HPLC chromatogram: (**A**) plasma and (**B**) brain samples at 30 min p.i of [^18^F]**BIT1** in a CD-1 mouse; (**C**) sample from incubation of **BIT1** with mouse liver microsomes (MLM) in the presence of NADPH for 90 min at 37 °C (UV detection: 228 nm; axis of retention time was adjusted). Conditions: isocratic system, column: Reprosil-Pur C18-AQ, 250 × 4.6 mm; eluent: 42% MeCN in 20 mM NH_4_OAc_aq._, flow: 1.0 mL/min.

The in vivo metabolism of [^18^F]**BIT1** was investigated in plasma and brain homogenate obtained from mice at 30 min p.i.. Prior to the analysis with RP-HPLC, the samples were treated with a mixture of MeCN/H_2_O (4:1, v/v) to precipitate the proteins. The recovery of radioactivity in plasma and brain samples was 96% and 99%, respectively. Intact tracer accounted for 43% and 78% of total activity in plasma and brain, respectively, indicating higher metabolic stability of [^18^F]**BIT1** in comparison to our previous PDE2A radioligands [^18^F]**TA3**, [^18^F]**TA4**, and [^18^F]**TA5 [23]**. As shown in the chromatograms (Figure 5), the two radio-metabolites ([^18^F]**M1** and [^18^F]**M2**) found in the brain were also observed in the corresponding plasma sample, indicating their ability to penetrate the blood-brain barrier. For further clarification, samples obtained as previously described in our in vitro metabolism study with **BIT1** using mouse liver microsomes [28] were investigated similarly by HPLC, but with UV detection (Figure 5C). On the basis of the in vitro metabolites **M1** and **M2**, both elucidated by LC-MS in the mentioned study, we can conclude about the brain penetrating radio-metabolites [^18^F]**M1** and [^18^F]**M2** as products of *N*-oxidation or *C*-hydroxylation. Besides, some of the more polar radio-metabolites detected in plasma could only tentatively be assigned as formed by mono-oxygenation, di-oxygenation, reduction, and demethylation, but were not investigated further.

### 2.3. In Vivo PET-MRI Studies of [^18^F]**BIT1**

**Figure 6:**
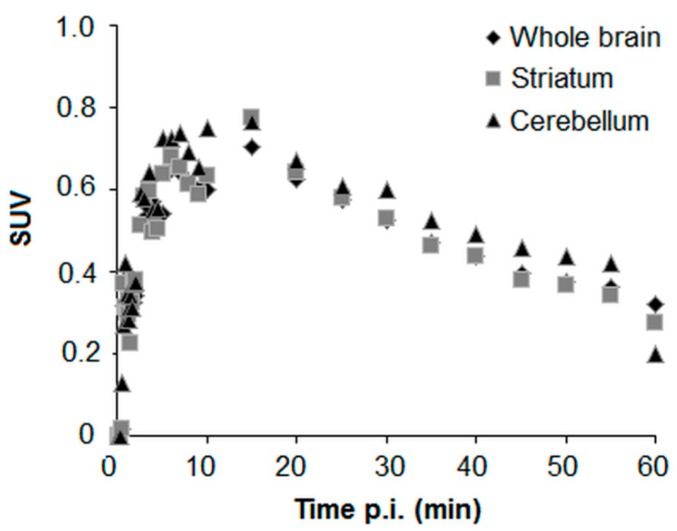
Averaged time-activity curves of [^18^F]**BIT1** in CD-1 mice (n = 2) with standard uptake values (SUV) in the whole brain, striatum, and cerebellum.

Dynamic PET-MRI studies were performed in female CD-1 mice after intravenous administration of [^18^F]**BIT1**. As reflected by the time-activity curves (TACs) shown in Figure 6, the radioactivity uptake in the whole brain, striatum, and cerebellum reached standardized uptake values (SUVs) of about 0.7 at 5 min p.i., indicating blood-brain barrier penetration. Since there is no difference in the TACs between the whole brain, striatum, and cerebellum, [^18^F]**BIT1** is assumed to possess low specific binding also in vivo. 

To further investigate this hypothesis, blocking studies were performed by concomitant injection of **TA1** and [^18^F]**BIT1**. However, since no significant reduction of radioactivity uptake in the PDE2A-specific region striatum was detectable (data not shown), the high non-specific binding of [^18^F]**BIT1** already observed in vitro was confirmed. 

Overall, the herein reported potential PDE2A radioligand, selected from a new class of 8-pyridinyl-BIT compounds, demonstrated sufficient blood-brain barrier (BBB) permeability. However, [^18^F]**BIT1** was found to be insufficient for in vivo imaging of PDE2A with PET. Further structural modifications are needed to obtain PDE2A selective radioligands for in vitro and in vivo research. Extensive structure-activity relationship (SAR) studies could lead to the improvement of the selectivity and specificity of compounds. In particular, modifications of the set substituents at 1- and 8-positions of the BIT scaffold might result in additive as well as nonadditive effects in compound potency [43].

## 3. Materials and Methods

### 3.1. General Methods

All chemicals were purchased from commercial sources and used without further purification. Solvents were dried before used if required. Air and moisture-sensitive reactions were carried out under argon atmosphere. Room temperature (RT) refers to 20–25 °C. Reaction mixtures were monitored by thin-layer chromatography (TLC) using pre-coated TLC-plates POLYGRAM^®^ SIL G/UV254 (Macherey-Nagel GmbH & Co. KG, Düren, Germany). The spots were detected under UV light at λ 254 nm and 365 nm. For the purification of final products, flash chromatography was performed with silica gel 40–63 μm (Merck KGaA, Darmstadt, Germany). Radio-TLC was performed on Polygram SIL G/UV_254_ plates (Macherey-Nagel GmbH & Co. KG, Düren, Germany) pre-coated plates with a mixture of chloroform/methanol (10:1, v/v) as eluent. The radio-TLC plates were exposed to storage phosphor screens (BAS-IP MS 2025, FUJIFILM Co., Tokyo, Japan) and recorded using the AmershamTyphoon RGB Biomolecular Imager (GE Healthcare Life Sciences, Freiburg, Germany), and the images were quantified with ImageQuant TL8.1 software (GE Healthcare Life Sciences, Freiburg, Germany).

NMR spectra (^1^H and ^13^C) were recorded on Mercury 300/Mercury 400 (Varian, Palo Alto, CA, USA) or Fourier 300/Avance DRX 400 Bruker (Billerica, MA, USA) instruments. Residual solvent proton (^1^H) and carbon (^13^C) resonances were used as internal standards for ^1^H-NMR (CDCl_3_, δ_H_ = 7.26; DMSO-*d_6_*, δ_H_ = 2.50) and ^13^C-NMR (CDCl_3_, δ_C_ = 77.16; DMSO-*d_6_*, δ_C_ = 39.52). The chemical shifts (δ) were reported in ppm as follows: s, singlet; d, doublet; t, triplet; m, multiplet, and the coupling constants (J) are reported in Hz. Mass spectra were recorded on an ESQUIRE 3000 Plus (ESI, low resolution) and a 7 T APEX II (ESI, high resolution) (Bruker Daltonics, Bruker Corporation, Billerica, MA, USA).

For semi-preparative HPLC, the following conditions were used. Column: Reprosil-Pur C18-AQ, 250 × 10 mm, particle size: 10 µm; eluent: 46% MeCN/20 mM NH_4_OAc_aq._; flow: 5.5 mL/min; ambient temperature; UV detection at 254 nm. The semi-preparative HPLC separations in the manual radiosynthesis were performed on a JASCO LC-2000 system, using a PU-2080-20 pump, an UV/VIS-2075 detector coupled with a radioactivity HPLC flow monitor (Gabi Star, raytest Isotopenmessgeräte GmbH, Straubenhardt, Germany) and a fraction collector (Advantec CHF-122SC, Dublin, CA, USA). Data recording was performed with the Galaxie chromatography software (Agilent Technologies, Santa Clara, USA).

Analytical chromatographic separations were performed on a JASCO LC-2000 system, incorporating a PU-2080*Plus* pump, AS-2055*Plus* auto-injector (100 µL sample loop), and a UV-2070 *Plus* detector coupled with a gamma radioactivity HPLC detector (Gabi Star, raytest Isotopenmessgeräte GmbH, Straubenhardt, Germany). Data analysis was performed with the Galaxie chromatography software (Agilent Technologies, Santa Clara, USA) using the chromatogram obtained at 254 nm. A Reprosil-Pur C18-AQ column (250 × 4.6 mm; 5 µm; Dr. Maisch HPLC GmbH, Ammerbuch-Entringen, Germany) with MeCN/20 mM NH_4_OAc_aq_. (pH 6.8) as eluent mixture and a flow of 1.0 mL/min was used (gradient: eluent A 10% MeCN/20 mM NH_4_OAc_aq_.; eluent B 90% MeCN/20 mM NH_4_OAc_aq_.; 0–10 min 100% A, 10–40 min up to 100% B, 40–45 min 100% B, 45–50 min up to 100% A, 50–60 min 100% A; isocratic system 42% MeCN/20 mM NH_4_OAc_aq_.; flow: 1.0 mL/min; ambient temperature).

The molar activities were determined on the basis of a calibration curve (0.1–6 µg of **BIT1**) performed under isocratic HPLC conditions (46% MeCN/20 mM NH_4_OAc_aq_.) using chromatograms obtained at 228 nm as the maximum of UV absorbance of compound **BIT1**.

No-carrier-added (n.c.a.) [^18^F]fluoride (t_1/2_ = 109.8 min) was produced by irradiation of [^18^O]H_2_O (Hyox 18 enriched water, Rotem Industries Ltd., Arava, Israel) via [^18^O(p,n)^18^F] nuclear reaction by irradiation of on a Cyclone^®^18/9 (iba RadioPharma Solutions, Louvain-la-Neuve, Belgium).

Remote-controlled automated syntheses were performed using a TRACERlab FX2 N synthesizer (GE Healthcare, USA) equipped with a N810.3FT.18 pump (KNF Neuburger GmbH, Freiburg, Germany), a BlueShadow UV detector 10D (KNAUER GmbH, Berlin, Germany), NaI(Tl)- counter, and the TRACERlab FX Software.

### 3.2. Precursor Synthesis and Radiochemistry

The final compounds described in this manuscript meet the purity requirement (>95%) determined by UV-HPLC.

#### 3.2.1. Synthesis of Precursor

*3-Methyl-8-(4,4,5,5-tetramethyl-1,3,2-dioxaborolan-2-yl)benzo[e]imidazo[5,1-c][1,2,4]triazine* (**2**)

A mixture of bromo derivative **1** [28] (1.32 g, 5 mmol), potassium acetate (1.1 g, 11.2 mmol), and bis(pinacolato)diboron (1.3 g, 5.11 mmol) in 2-MeTHF (20 mL) was degassed with argon for 10 min. After the addition of Pd(dppf)Cl_2_ (0.055 g, 0.075 mmol), the mixture was refluxed at 100 °C for 6 h and at 85 °C for 12 h. Upon completion of the reaction (monitored by TLC), CH_2_Cl_2_ (25 mL) was added, and the reaction mixture was stirred for 1 h. The solid was filtered off, and the filtrate was evaporated. The dark brown semi-solid residue (2.5 g) was dissolved in MTBE (60 mL) and filtered through a short silica gel column (H: 3 cm × D: 2 cm). Heptane (30 mL) was added to the eluate, followed by concentration to a volume of approximately 20 mL. The precipitated solid was filtered and dissolved in MTBE (120 mL), and the solution obtained was filtered again through a short silica gel column (H: 2 cm × D: 2 cm). The yellow eluate was concentrated (→ ~15 mL) and treated with *n*-heptane (30 mL). The solid obtained was filtered and dried under reduced pressure to afford a yellow solid **2** (1.32 g, 85%). TLC [CHCl_3_/MeCN (10:1)]: R_f_ = 0.26 (strong tailing). ^1^H NMR (400 MHz, CDCl_3_) δ_H_ = 8.57 (s, 1H), 8.41 (d, J = 8.0 Hz, 1H), 8.25 (s, 1H), 8.05 (dd, J = 8.0, 1.1 Hz, 1H), 2.89 (s, 3H), 1.40 (s, 13H).

*3-Methyl-8-(2-nitropyridin-4-5 yl)benzo[e]imidazo[5,1-c][1,2,4]triazine* (**3**)

A mixture of boronic ester **2** (470 mg, 1.5 mmol, 1 eq) and 4-bromo-2-nitropyridine (243 mg, 1.2 mmol) in 1,2-dimethoxyethane (6 mL) and an aqueous solution of K_2_CO_3_ (2 M, 1.75 mL, 3.5 mmol) was degassed with argon for 20 min. Pd(PPh_3_)_4_ (50 mg, 0.043 mmol) was added, and the reaction mixture was heated at 95 °C for 1 h and 88 °C for 14 h. Upon full conversion of starting material (monitored by TLC), the solvent was evaporated, and the residue was partitioned between water (30 mL) and CHCl_3_ (50 mL). After separation of the organic layer, the aqueous layer was extracted with CHCl_3_/MeCN (8:1, 6 × 30) and CHCl_3_ (5 × 30 mL). The combined organic layers were dried over Na_2_CO_3_, and the solvent was evaporated to leave a solid residue (370 mg). The residue was refluxed in MeCN (50 mL) for 5 min, and after cooling to 0–2 °C, the product was filtered and dried to give **3** as yellow solid (233 mg, 63%). TLC [CHCl_3_/MeOH/30% NH_3_ (10:1:0.1)]: R_f_ = 0.35. ^1^H NMR (300 MHz, DMSO-d6) δ_H_ = 9.26 (s, 1H), 8.96 (dd, J = 2.0, 0.5 Hz, 1H), 8.85 (dt, J = 5.1, 0.8 Hz, 1H), 8.82–8.80 (m, 1H), 8.52 (dd, J = 8.7, 1.5 Hz, 1H), 8.42 (dd, J = 5.1, 1.7 Hz, 1H), 8.28 (dd, J = 8.5, 2.0 Hz, 1H), 2.78 (d, J = 1.7 Hz, 3H). ^13^C NMR (75 MHz, DMSO-d6) δ_C_ = 157.67, 149.59, 149.28, 138.82, 138.14, 136.18, 135.67, 130.31, 127.22, 127.17, 126.02, 122.45, 115.55, 114.35, 12.18.

*1-Brom-3-methyl-8-(2-nitropyridin-4-yl)benzo[e]imidazo[5,1-c][1,2,4]triazine* (**4**)

*N*-Bromosuccinimide (NBS) (48 mg, 1.58 eq) was added to a suspension of compound **3** (52 mg, 1 eq) in 2.5 mL MeCN. The reaction mixture was protected from light and stirred at RT for 1–2 d. After the complete conversion of starting material (monitored by TLC), the mixture was partitioned between CHCl_3_ (15 mL) and water (15 mL). The organic layer was washed with aqueous NaHCO_3_ (5%, 15 mL) and Na_2_SO_3_ (5%, 15 mL). The aqueous layer was extracted again with CHCl_3_ (4x5 mL). The combined organic layers were dried (Na_2_SO_4_) and evaporated. The crude product was purified by column chromatography (CHCl_3_/MeOH, 20:1) to afford compound **4** as a yellow solid (64 mg, 97%). TLC [CHCl_3_/MeOH (20:1)]: R_f_ = 0.59. ^1^H NMR (400 MHz, CDCl_3_) δ_H_ = 9.31 (d, *J* = 1.8 Hz, 1H), 8.84 (d, *J* = 5.0 Hz, 1H), 8.65 (d, *J* = 8.4 Hz, 1H), 8.59 (d, *J* = 1.6 Hz, 1H), 8.04–7.98 (m, 2H), 2.90 (s, 3H).

*1-(2-Chloro-5-methoxyphenyl)-3-methyl-8-(2-nitropyridin-4-yl)benzo[e]imidazo[5,1-c][1,2,4]triazine* (**5**)

Brominated compound **4** (43 mg, 0.112 mmol, 1 eq), 2-chloro-5-methoxyphenyl boronic acid (27 mg, 0.145 mmol, 1.29 eq), and K_2_CO_3_ (44 mg, 0.318 mmol, 2.8 eq) were combined in a mixture of 1,4-dioxane and H_2_O (4:1). Under argon atmosphere, [Pd(PPh_3_)_4_] (13 mg, 10 mol%) was added. After refluxing at 101–105 ^°^C for 3 h, the reaction mixture was stirred at RT for 16 h. Afterward, the residue was partitioned between CHCl_3_ (20 mL) and water (20 mL). The organic layer was washed with water (20 mL) and brine (20 mL). The aqueous layer was extracted with CHCl_3_ (3 × 10 mL), and the combined organic layers were dried over Na_2_SO_4_, and the solvent was evaporated under reduced pressure. The crude product was purified by column chromatography using a gradient system (*n*-hexane/ethyl acetate from 1:1 to 1:3) to get the corresponding precursor **5** as a yellow solid (34 mg, 68%). TLC [*n*-hexane/ethyl acetate (1:2)]: R_f_ = 0.38. ^1^H NMR (400 MHz, CDCl_3_) δ_H_ = 8.71 (dd, *J* = 5.0, 0.6 Hz, 1H), 8.60 (d, *J* = 8.4 Hz, 1H), 8.15 (dd, *J* = 1.6, 0.6 Hz, 1H), 7.95 (dd, *J* = 8.4, 1.9 Hz, 1H), 7.77 (dd, *J* = 5.0, 1.6 Hz, 1H), 7.67–7.64 (m, 1H), 7.58 (d, *J* = 1.9 Hz, 1H), 7.24–7.20 (m, 2H), 3.87 (s, 3H), 3.00 (s, 3H). ^13^C NMR (101 MHz, CDCl_3_) δ_C_ = 159.24, 157.85, 150.52, 149.96, 140.32, 138.66, 137.53, 137.22, 136.47, 131.76, 131.27, 131.11, 126.50, 125.79, 125.61, 124.31, 118.59, 117.99, 115.77, 114.21, 56.06, 12.94. ESI (+): *m/z* = 447.10 (calcd. 447.10 for C_22_H_16_ClN_6_O_3_^+^ [M + H]^+^).

#### 3.2.2. In Vitro Metabolism of **BIT1**

**BIT1** was incubated with mouse liver microsomes in the presence of NADPH, including positive and negative controls, for 90 min at 37 °C, as previously described [28]. Obtained samples were investigated by analytical HPLC with an isocratic system (42% MeCN/20 mM NH_4_OAc_aq_; flow rate of 1.0 mL/min) and UV detection (228 nm).

#### 3.2.3. Radiochemistry

##### Manual Radiosynthesis

No carrier added [^18^F]fluoride in 1.5 mL water and was trapped on a Chromafix^®^ 30 PS-HCO_3_^−^ cartridge (Macherey-Nagel GmbH & Co. KG, Düren, Germany). The activity was eluted with 300 μL of an aqueous solution of potassium carbonate solution (K_2_CO_3_, 1.8 mg, 13 μmol) into a 4 mL V-vial, and Kryptofix_2.2.2_. (K_2.2.2_) (11 mg, 29 μmol) in 1.0 mL MeCN was added. The aqueous [^18^F]fluoride was azeotropically dried under vacuum and nitrogen flow within 7–10 min using a single-mode microwave (75 W, at 50–60 °C, power cycling mode). Two aliquots of MeCN (2 × 1.0 mL) were added during the drying procedure, and the final complex was dissolved in 500 µL of solvent (DMSO, DMF, and MeCN) ready for labeling. Thereafter, 0.5–1.0 mg of precursor in 500 µL labeling solvent was added, and the ^18^F-labeling was performed at different temperatures (100 °C, 120 °C, and 150 °C). To analyze the reaction mixture and to determine radiochemical yields, samples were taken for radio-HPLC and radio-TLC at different time points (5, 10, 15, and 20 min).

After cooling to <30 °C, the reaction mixture was diluted with 1.0 mL water and 2.0 mL of MeCN/H_2_O (1:1) and directly applied to an isocratic semi-preparative RP-HPLC for isolation of [^18^F]**BIT1** (46% MeCN/20 mM NH_4_OAc_aq._, 5.5 mL/min, Reprosil-Pur C18-AQ column, 250 × 10 mm; 10 µm; Dr. Maisch HPLC GmbH, Ammerbuch-Entringen, Germany). The collected radiotracer fraction was diluted with 35 mL water to perform purification by sorption on a Sep-Pak^®^ C18 light cartridge (Waters, Milford, MA, USA) and successive elution with 0.75 mL of ethanol.

##### Automated Radiosynthesis of [^18^F]**BIT1**

**Figure 7:**
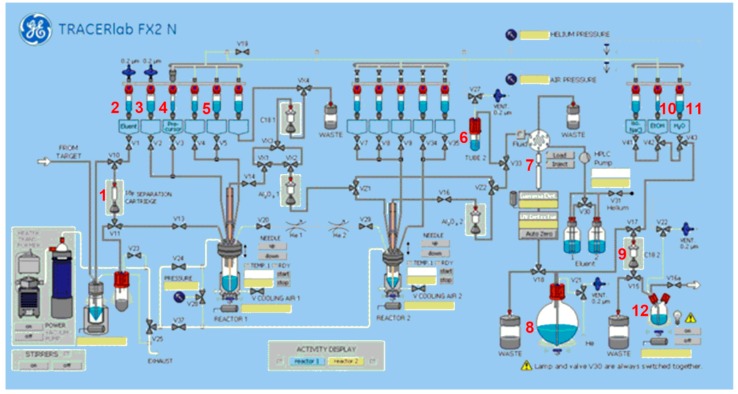
Scheme of the synthesis module TRACERlab FX2 N for the radiosynthesis of [^18^F]**BIT1**. (1) Chromafix^®^ 30 PS-HCO_3_−, (2) K_2_CO_3_ (1.8 mg in 400 μL water), (3) K_2.2.2._ (11 mg, 29 µmol in 1.0 mL MeCN), (4) precursor (0.5 mg of **5** in 1.0 mL DMSO), (5) 1.0 mL H_2_O and 2.0 mL MeCN/H_2_O (1:1), (6) injection vial, (7) Reprosil-Pur C18-AQ column (46% MeCN/20mM NH_4_OAc_aq_., flow 5.5 mL/min), (8) 35 mL water, (9) Sep-Pak^®^ C18 light, (10) 2.0 mL water, (11) 1.2 mL EtOH, (12) product vial.

The automated radiosynthesis was performed in a TRACERlab FX2 N synthesis module (GE Healthcare, Waukesha, WI, USA). About 2–4.5 GBq of aqueous n.c.a [^18^F]fluoride was trapped on a Chromafix^®^ 30 PS-HCO_3_^-^ cartridge (Figure 7, Macherey-Nagel GmbH & Co. KG, Düren, Germany, entry 1) and eluted with K_2_CO_3_ (1.8 mg, 13 µmol, entry 2) in 400 µL water. Kryptofix K_2.2.2_ (11 mg, 29 μmol, in 1.0 mL MeCN, entry 3) was directly added. Afterward, azeotropic drying was performed at 60 °C and 85 °C under a vacuum. Thereafter, 0.5 mg of **5** in 1.0 mL DMSO (entry 4) was transferred to the reactor, and subsequently, the reaction was performed at 100 °C for 5 min. After cooling the reactor to 30 °C, 1.0 mL of H_2_O and 2.0 mL of MeCN/H_2_O (1:1) (entry 5) were added and transferred into the injection vial (entry 6). The semi-preparative HPLC was performed using a Reprosil-Pur C-18 AQ column (250 × 10 mm; 10 µM) using 46% MeCN in aqueous 20 mM ammonium acetate at a flow rate of 5.5 mL/min (entry 7). The [^18^F]**BIT1** fraction was pooled in 35 mL H_2_O (entry 8) and then loaded on a preconditioned Sep-Pak^®^ C18 light cartridge (Waters, Milford, MA, USA, entry 9), and washed with 2.0 mL of H_2_O (entry 10). Afterward, the trapped [^18^F]**BIT1** was eluted with 1.2 mL EtOH (entry 11) into the product vial (entry 12). The product was transferred out of the hot cell, and the solvent was reduced under a gentle argon stream at 70 °C to a final volume of 10–50 μL. Afterward, the radiotracer was diluted in isotonic saline to obtain a final product containing 10% of EtOH (v/v). The identity of the final product was confirmed using radioanalytical HPLC.

The molar activity of [^18^F]**BIT1** was determined by analytical HPLC (isocratic mode: 46% MeCN/20 mM NH_4_OAc_aq._, Reprosil-Pur C18-AQ column 250 × 4.6 mm, flow rate: 1.0 mL/min). The UV/Vis detection was carried out at 228 nm as the absorption maximum of **BIT1**.

##### Determination of Stability

In vitro stability of [^18^F]**BIT1** was investigated by incubation of the radioligand in phosphate-buffered saline (PBS, pH 7.4), *n*-octanol, and pig plasma at 40 °C for 60 min (~5 MBq of the radioligand was added to 500 μL of each medium). Samples were taken at 30 and 60 min and analyzed by radio-TLC and radio-HPLC.

##### Determination of log D

The lipophilicity of [^18^F]**BIT1** was determined by partitioning between *n*-octanol and phosphate-buffered saline (PBS, pH 7.4) at ambient temperature using the conventional shake-flask method. An aliquot of 10 μL of the formulated solution containing ~500 kBq of [^18^F]**BIT1** was added to a tube containing 6 mL of the *n*-octanol/PBS-mixture (1:1, v/v, four-fold determination). The tubes were shaken for 20 min using a mechanical shaker (HS250 basic, IKA Labortechnik GmbH & Co. KG, Staufen, Germany) followed by centrifugation (5000 rpm for 5 min) and separation of the phases. Aliquots of 1 mL of the organic and the aqueous phase were taken, and the activity was measured using an automated gamma counter (1480 WIZARD, Fa. Perkin Elmer, Waltham, MA, USA). The distribution coefficient (D) was calculated as [activity (cpm/mL) in *n*-octanol]/[(activity (cpm/mL) in PBS], specified as the decadic logarithm (logD).

### 3.3. Animal Studies

All experimental work, including animals, was conducted in accordance with the national legislation on the use of animals for research (Tierschutzgesetz (TierSchG), Tierschutz-Versuchstierverordnung (TierSchVersV)) and was approved by the responsible research ethics committee (TVV 18/18, DD24.1-5131/446/19, Landesdirektion Sachsen, 20th June 2018). Female CD-1 mice, 10–12 weeks, were obtained from the Medizinisch-Experimentelles Zentrum at Universität Leipzig. For the time of the experiments, the animals were kept in a dedicated climatic chamber with free access to water and food under a 12:12 h dark:light cycle at a constant temperature of 24 °C. Piglet brains were obtained from anesthetized and euthanized juvenile female German landrace pigs (Lehr- und Versuchsgut Oberholz, Universität Leipzig).

#### 3.3.1. In Vitro Autoradiography of [^18^F]**BIT1**

Pig brain cryosections (16 µm; Microm HM560 Cryostat, FischerScientific GmbH, Schwerte, Germany) were thawed, dried in a stream of cold air, and preincubated for 10 min with buffer (50 mM TRIS-HCl, pH 7.4, 120 mM NaCl, 5 mM KCl, 2 mM MgCl_2_) at room temperature. Afterward, brain sections were incubated with 1.72 nM of [^18^F]**BIT1** in the buffer for 60 min at room temperature. The sections were then washed twice with ice-cold 50 mM TRIS-HCl (pH 7.4), dipped in ice-cold deionized water, dried in a stream of cold air, and exposed for 60 min to an image plate. The analysis was performed using an image plate scanner (HD-CR 35; Duerr NDT GmbH, Bietigheim Bissingen, Germany). Non-specific binding was determined using 10 µM **TA1** and **BIT1** as blocking compounds. 

#### 3.3.2. In Vivo Metabolism of [^18^F]**BIT1**

[^18^F]**BIT1** (~19 MBq) was injected in female CD-1 mice (10–12 weeks old) via the tail vein. Brain and blood samples were obtained at 30 min p.i., plasma separated by centrifugation (14,000 rpm, 1 min), and brain homogenized in ~1 mL isotonic saline on ice (10 strokes of a PTFE plunge at 1000 rpm) in borosilicate glass.

Protein precipitation of plasma and brain samples was performed in duplicate with ice-cold MeCN/H_2_O (8:2, v/v), which was added to the samples (sample/solvent, 1:4, v/v). The samples were vortexed for 1 min, followed by resting on ice for 10 min. Afterward, the samples were centrifuged for 5 min at 10,000 g. Supernatants were collected, and 100 µL ice-cold MeCN/H_2_O (8:2, v/v) was added to the pellets for the second extraction, applying the same treatment as before. The combined supernatants were concentrated at 70 °C under nitrogen stream until a remaining volume of 100 µL and subsequently quantified by analytical radio-HPLC with an isocratic system (42% MeCN/20 mM NH_4_OAc_aq_.; flow rate: 1.0 mL/min). The activity recovery was determined by measuring the radioactivity of aliquots taken from supernatants and the pellets using a gamma counter.

### 3.4. PET-MRI Studies of [^18^F]**BIT1**

PET/MRI scans were performed using a preclinical PET/MRI system (nanoScan, Mediso Medical Imaging Systems, Budapest, Hungary). Animals were anesthetized with a mixture of air/isoflurane (2.0%, 200 mL/min) (Anesthesia Unit U-410, agntho’s, Lidingö, Sweden), and their body temperature maintained at 37 °C with a thermal bed system. [^18^F]**BIT1** (2 MBq, A_m_: 155 GBq/µmol, EOS) was injected via the tail vein, either without (baseline study, n = 2) or with **TA1** (2 mg/kg, DMSO/NaCl (1:25); blocking study, n = 2). A dynamic 60-min PET scan was started 20 sec before radioligand injection. The list-mode data were reconstructed into 33 frames (12 × 10 sec, 6 × 30 sec, 5 × 1 min, 10 × 5 min) with 3D-ordered subset expectation maximization, 4 iterations, and 6 subsets, using an energy window of 400 to 600 KeV and coincidence mode of 1–5. After the PET scan, an MRI scan was performed for anatomical orientation and attenuation correction on a 1T magnet using a T1-weighted gradient-echo sequence (TR = 15 ms, TE = 2.6 ms). The data analysis was done with PMOD (PMOD Technologies Ltd., Zurich, Switzerland, v. 3.6), and an atlas-based method was used to obtain SUV time-activity curves for the striatum, cerebellum, and the whole brain.

## 4. Conclusions

In our efforts to develop a specific radioligand for PET imaging of PDE2A in the brain on the basis of a benzoimidazotriazine scaffold, we successfully prepared the novel PDE2A radioligand [^18^F]**BIT1** with a high radiochemical yield, radiochemical purity, as well as molar activity. Our findings showed that this class of compound demonstrated good brain penetration and sufficient in vivo metabolic stability. However, [^18^F]**BIT1** was not suitable for the molecular imaging of PDE2A in the brain because of a high non-specific binding. Further structural modifications are required to obtain more satisfactory PDE2A specific radioligands.

## Supplementary Materials

The following are available online, Figures S1–S2: NMR spectra of precursor **5** (^1^H and ^13^C), Figure S3. The calibration curve of molar activity of reference **BIT1**.
