# Supplementary material for: Radiosynthesis and Biological Investigation of a Novel Fluorine-18 Labeled Benzoimidazotriazine-Based Radioligand for the Imaging of Phosphodiesterase 2A with Positron Emission Tomography
Source: Molecules. 2019 Nov 15;24(22):4149. doi: 10.3390/molecules24224149 (PMC6891464; doi:10.3390/molecules24224149)
Supplement: Supplementary file 1 [file molecules-24-04149-s001.pdf]

molecules

Supplementary Information

Radiosynthesis and Biological Investigation of a Novel Fluorine-18 Labeled Benzoimidazotriazine-Based Radioligand for Imaging of Phosphodiesterase 2A with Positron Emission Tomography

Rien Ritawidya [1,2,*], Barbara Wenzel [1], Rodrigo Teodoro [1], Magali Toussaint [1], Mathias Kranz [3,4], Winnie Deuther-Conrad [1], Sladjana Dukic-Stefanovic [1], Friedrich-Alexander Ludwig [1], Matthias Scheunemann [1], and Peter Brust [1]

[1] Helmholtz-Zentrum Dresden-Rossendorf, Institute of Radiopharmaceuticals Cancer Research, Research Site Leipzig, Department of Neuroradiopharmaceuticals, Leipzig 04318, Germany
[2] Center for Radioisotope and Radiopharmaceutical Technology, National Nuclear Energy Agency (BATAN), Puspiptek Area, Serpong, South Tangerang 15314, Indonesia
[3] Tromsø PET Centre, University Hospital of North Norway, 9009 Tromsø, Norway
[4] Nuclear Medicine and Radiation Biology Research Group, The Arctic University of Norway, 9009 Tromsø , Norway
* Correspondence: r.ritawidya@hzdr.de or rienrita@batan.go.id
Tel.: +49-341-234-179-4611 or +62-21-756-3141

Table of Contents

Figure S1. ^{1}H NMR (400 MHz, $CDCl_3$) spectrum of precursor **5** **S3**
Figure S2. ^{13}C NMR (101 MHz, $CDCl_3$) spectrum of precursor **5** **S4**
Figure S3. The calibration curve of molar activity of reference **BIT1** **S5**

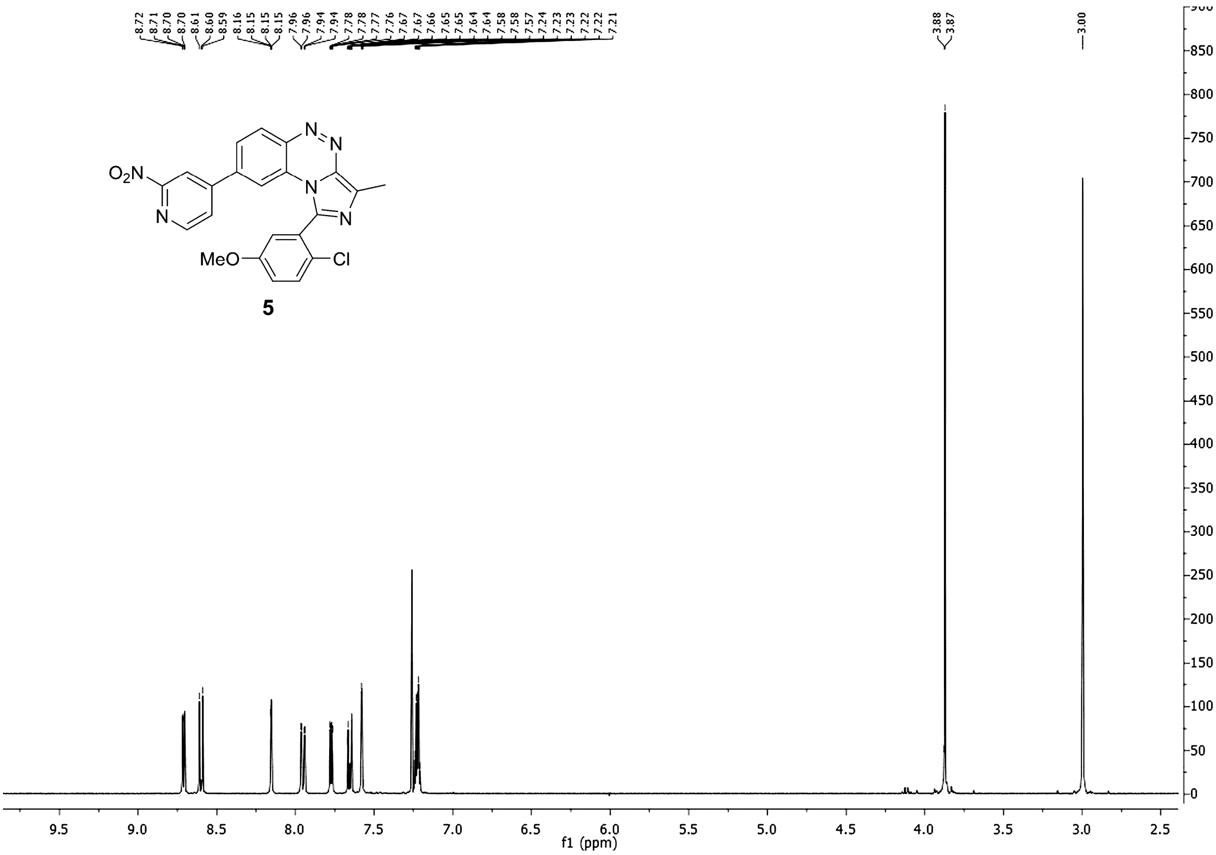

Figure S1. ^{1}H NMR (400 MHz, $CDCl_3$) spectrum of precursor **5**

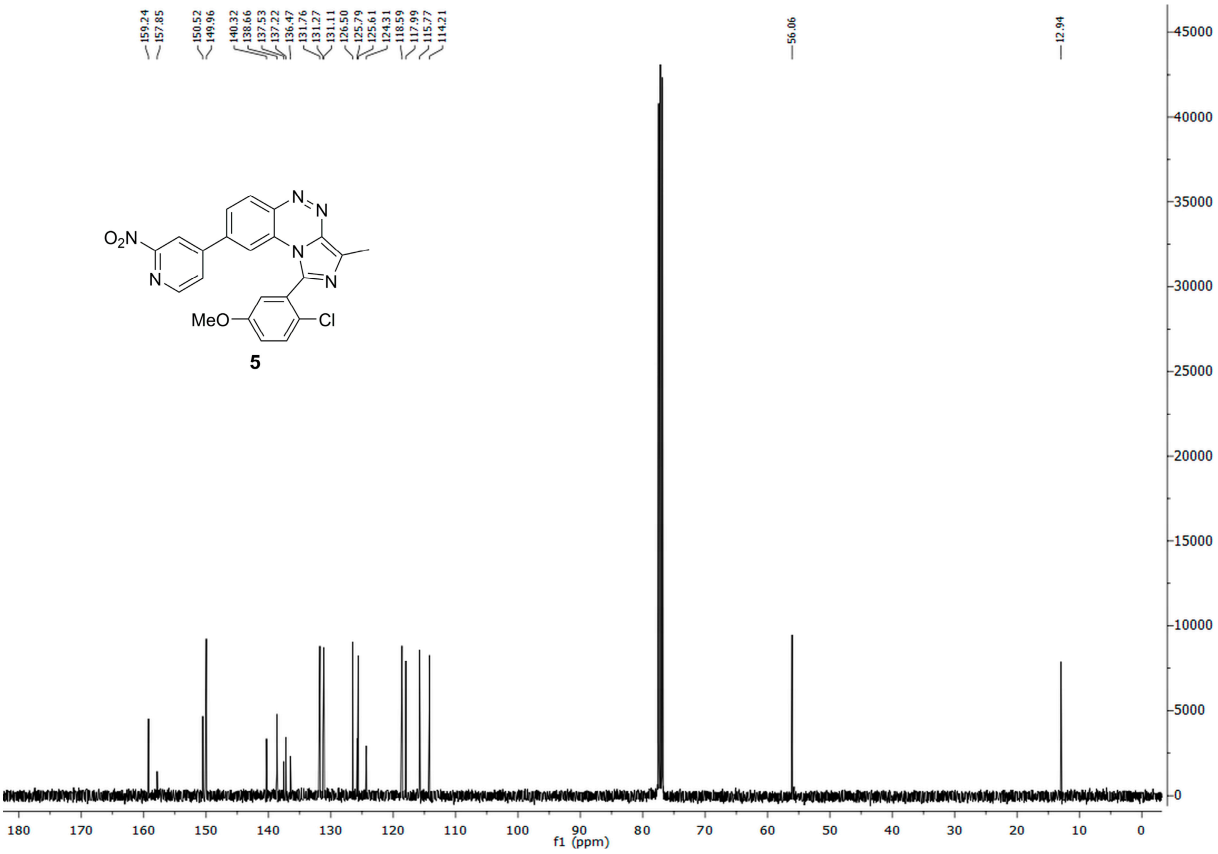

Figure S2. ^{13}C NMR (101 MHz, $CDCl_3$) spectrum of precursor **5**

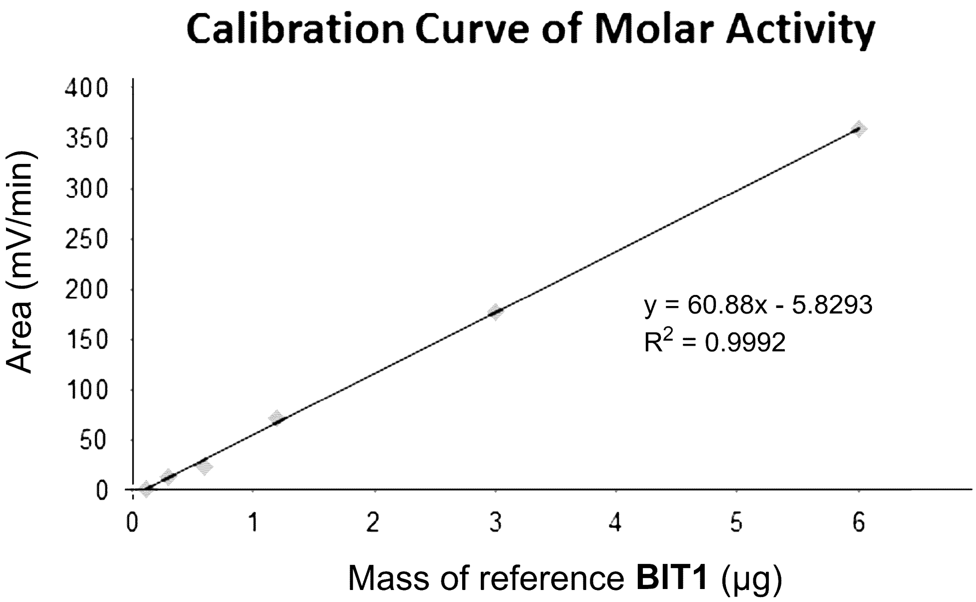

Figure S3. The calibration curve of molar activity of reference **BIT1**
